# Severity, symptomatology, and treatment duration for mental health disorders: a retrospective analysis from a conflict-affected region of northern Nigeria

**DOI:** 10.1186/s13031-022-00473-x

**Published:** 2022-07-15

**Authors:** Santiago Martínez Torre, Cristina Carreño, Luis Sordo, Augusto E. llosa, Janet Ousley, Abdulrauf Waziri, Richard Mathela, Retsat Dazang Umar, Joshua Usman, María José Sagrado

**Affiliations:** 1 Médecins Sans Frontières, Carrer de Zamora, 54, 08005 Barcelona, Spain; 2 Department of Public Health and Child Health, Faculty of Medicine, Complutense University, Madrid, Spain; 3 Consortium for Biomedical Research in Epidemiology and Public Health (CIBERESP), Madrid, Spain; 4 Médecins Sans Frontières, Paris, France; 5 Médecins Sans Frontières, Abuja, Nigeria

**Keywords:** MHPSS, Armed conflict, Borno State, CGI-S scale, Therapeutic duration, Humanitarian

## Abstract

**Background:**

Mental Health and psychosocial support (MHPSS) programs are essential during humanitarian crises and in conflict settings, like Nigeria’s Borno State. However, research on how types of traumatic stress and symptom severity affect clinical improvement is lacking in these contexts, as is consensus over how long these patients must engage in mental health care to see results.

**Methods:**

Records from 11,709 patients from the MHPSS program in Pulka and Gwoza local government areas in Borno State, Nigeria from 2018 and 2019 were retrospectively analyzed. Patient information, symptoms, stress type, severity (CGI-S scale), and clinical improvement (CGI-I and MHGS scales) were assessed by the patient and counselor. Associations between variables were investigated using logistic regression models.

**Results:**

Clinical improvement increased with consultation frequency (OR: 2.5, *p* < 0.001 for CGI-I; OR: 2, *p* < 0.001 for MHGS), with patients who received three to six counseling sessions were most likely to improve, according to severity. Survivors of sexual violence, torture, and other conflict/violence-related stressors were nearly 20 times as likely to have posttraumatic stress disorder (PTSD) (OR: 19.7, *p* < 0.001), and depression (OR: 19.3, *p* < 0.001) symptomatology. Children exposed to conflict-related violence were also almost 40 times as likely to have PTSD (OR: 38.2, *p* = 0.002). Most patients presented an improvement in outcome at discharge, per both counselors (92%, CGI-I) and self-rating scores (73%, MHGS).

**Conclusion:**

We demonstrate a threshold at which patients were most likely to improve (3 sessions for mild or moderate patients; 6 sessions for severe). In addition, we identify the specific types of stress and symptom severity that affected the number of sessions needed to achieve successful outcomes, and highlight that some stress types (especially torture or having a relative killed) were specifically linked to PTSD and depression. Therefore, we emphasize the importance of classifying patient stress type and severity to identify the appropriate duration of care needed.

## Introduction

An increase in conflict-related violence in northern Nigeria in recent years has had a substantial impact on Borno State, causing more than 2 million people to be displaced and thousands of deaths. Since late 2014, there have been nearly daily attacks on civilians by rebel groups. Pulka and Gwoza are two villages located 18 km apart. Pulka is an enclave surrounded by military forces, where around 63,000 people live, 28,000 of whom are internally displaced persons (IDPs) living in five IDPs camps. Gwoza ´s population is around 62,000 people, with 11,000 IDPs integrated into a host community (41,000 people) and three IDPs or temporary camps. Both have been at the center of the conflict with non-state armed groups, experiencing violence, insecurity, civilian killings, forced displacement, abduction of women and men, sexual slavery of women and girls, and forced disappearance [1].

Yet, response to violence varies by individuals and communities. Some can face extreme stress, especially when strong community support and coping mechanisms are present, whereas others will be affected by mental health (MH) and psychosocial disorders. It is estimated in conflict settings that MH and psychosocial disorders may affect as much as 22.1% of the population, among whom 5.1% will present with a moderate-severe MH condition, most commonly posttraumatic stress [PTSD], depression, or anxiety disorders [2–6]. Other severe mental disorders, such as psychosis, are less prevalent but have a more deleterious impact on patients and communities, occurring with a higher number of comorbidities, reduced life expectancy of around 10–20 years, and high socioeconomic consequences [7]. Potentially traumatic events (PTEs) are also associated with specific MH disorders: torture, witnessing a murder or physical abuse, receiving threats, or suffering property destruction and loss, are associated with PTSD [4]. Chronic PTE exposure is associated with depression [8]. Counseling programs in humanitarian settings often see a high prevalence of anxiety and mood-related complaints, with conflict and violence (especially when domestic or sexual abuse was involved) being some of the most powerful triggers [5].

Mental Health and psychosocial support (MHPSS) programs are essential during humanitarian crises and conflict settings like Borno and are often integrated into broader health care programs [9]. While some evidence has examined the positive impact of brief MHPSS interventions in humanitarian contexts [6, 10], there is a paucity of research examining field realities in active clinical sites. Moreover, a better understanding of the relationship between MH patients’ disease severity, their improvement during and after care, the overall impact of MHPSS, and the number of consultations needed to reach results are all needed [11–13]. Here we describe clinical features, sociodemographic characteristics, and intervention outcomes from patients treated in an MHPSS program in a conflict-affected area of northern Nigeria. We attempt to better understand how traumatic stress and duration in MH care influences patients’ improvement in conflict settings.

## Materials and methods

This retrospective study used routinely collected clinical data from patients presenting for MHPSS services at facilities supported by Médecins Sans Frontières (MSF) in Pulka and Gwoza Local Government Areas (LGA) in Borno State, Nigeria, from January 2018 to December 2019. MSF MHPSS activities are part of a humanitarian medical program that provides primary and secondary health care services in health facilities and in the community. MSF MHPSS services included psychological and pharmacological care, counseling, focused psychosocial support groups, psychosocial stimulation, psychoeducation, recreational activities, and psychological first aid (PFA). MHPSS activities were delivered by community MH workers and lay counselors trained and supervised by clinical psychologists. Patients with severe MH disorders were managed by a medical doctor trained in the World Health Organization (WHO) Mental Health Gap Action Programme (mhGAP) intervention approach as well as by a clinical psychologist, with remote supervision provided by a psychiatrist [14].

Eight mental, neurological, and substance use (MNS) symptom categories were developed using a consultative process with MSF experts, the International Disease Classification 10 (ICD-10) manual, and the Diagnostic and Statistical Manual of Mental Disorders-IV (DSM-IV). MNS symptoms categories included: (1) somatoform symptoms, (2) anxiety-related symptoms, (3) posttraumatic symptoms, (4) depression-related symptoms, (5) psychosis-related symptoms, (6) behavioral symptoms, (7) cognitive symptoms, and (8) other symptoms. Each category included subcategories with specific symptoms that facilitated classification (though subcategories were not used for study purposes). Usually, more than one category was registered. Counselors recorded the patient’s primary symptoms and listed them severity (“Symptom 1,” “Symptom 2,” and “Symptom 3”). “Symptom 1” corresponded to a patient´s predominant symptom and determined their primary symptom category. In addition to symptoms, the counselor also recorded stress types that contributed to symptoms.

Data were collected from patient charts by an attending counselor after each session. All data used in the study were routinely collected for program monitoring purposes. Patients’ enrollment date, clinical and sociodemographic characteristics, and types of stress they experienced were recorded. A clinical evaluation was conducted to document symptoms and severity. Confidentiality was protected throughout, and all data was de-identified.

Severity was measured by counselors using the Clinical Global Impression-Severity scale (CGI-S) at enrollment and categorized according to the instrument’s 7-point scale: 0 (not assessed), 1 (normal, not at all ill), 2 (borderline mentally ill), 3 (mildly ill), 4 (moderately ill), 5 (markedly ill), 6 (severely ill) and 7 (among the most extremely ill patients). Severity was also measured using the Mental Health Global State (MHGS) scale, based on how a patient’s symptoms interfered with their everyday living and impact their functionality. If the patient was ≤ 15 years old, the MHGS scale ranged from 1 to 65 points based on 14 questions, depending on severity. If the patient was > 15 years old, the MHGS scale ranged from 1 to 30 points, based on 6 questions. Improvement was measured by counselors at every session using two scales: The Clinical Global Impression-Improvement (CGI-I) scale and the MHGS scale variation. An outcome was categorized as “improvement” according to values in each scale: a difference between enrollment and the final consultation of 4 points for adults and 7 points for children following the MHGS scale variation, and 1 (“very much improved”), 2 (“much improved”), or 3 (“minimally improved”) points following the CGI-I scale. The CGI scale is a universal tool routinely used in research and clinical practice, and the MHGS scale was developed by MSF for use in humanitarian contexts and has demonstrated cross-cultural utility [15–18].

### Statistical analysis

We analyzed data from patients who received MH care and had participated in at least one MH consultation. Analysis was disaggregated by sex and age group. Variables were summarized using percentages or means, standard deviations (SD) or medians, and inter-quartile ranges (IQR), as appropriate. We compared values using the Student's t-test, Wilcoxon Mann–Whitney testing, as appropriate, or Pearson's chi-squared testing, setting statistical significance at *p* < 0.05. To measure the expected associations between variables, univariable logistic regression models were fitted and odds ratios (OR) presented with corresponding 95% confidence intervals (CI) and *p* values. Subsequently, all variables were introduced and explored into multivariable logistic regression crude models. Statistically significant variables (*p* < 0.05) were selected for a final logistic regression model measuring the associations between patient characteristics and outcomes, and OR were presented with their corresponding 95% CI and *p* values. Multicollinearity has been considered using the VIF test for multicollinearity. Subsequently, we have determined which variable to remove, by creating a correlation matrix to view the correlation coefficients between each of the variables in the model. The following goodness of fit statistic was considered for all models: Pearson's Goodness-of-Fit Test (acceptable model fit if *p* > 0.05). Pseudo R^2^ was considered for all models as a measure of variance (range from 0 to 1). Descriptive, univariable, and multivariable analyses were stratified or adjusted by age group (children ≤ 15 years old and adults > 15 years old) and gender. Finally, we estimated the average predicted probability of improvement by the number of consultations using margins of responses, obtained from logistic regression model predictions that were based on CGI-I and MHGS scales (given the input variable number of consultations). Analysis was performed with STATA SE v15.

### Ethics

As this study used routine programmatic data and took the necessary steps to protect patient confidentiality, it was exempted from full review by the MSF Ethical Review Board and the National Health Research Ethics Committee of Nigeria (NHREC). All study procedures were performed in accordance with the Declaration of Helsinki.

## Results

Data were collected from 11,709 patient files from January 2018 to December 2019. Patients without a final consultation, (defined as those whose files were not ‘closed’ because they were lost to follow-up (LTFU) or who participated in only a single session (n = 5663) were excluded from outcome analysis (n = 6046 patients analyzed). The male to female ratio was 0.3 among the 11,709 participants with baseline information. Most patients were female (77.9% [0.77–0.79]; *p* = 0.03; n = 9100) as opposed to male (22.1% [0.21–0.23]; *p* = 0.03; n = 2580). Mean (SD) age at enrollment was 32.7 (13.1), and 11.7% of the patients (n = 1365) were < 15 years of age. The male to female ratio among children was 1.1. A large majority (70.5%; n = 8254) had been forcibly displaced. 73.9% of patients were illiterate (n = 8647) (Table 1).

**Table 1:** Baseline characteristics of the population

	Male (n = 2585)	Female (n = 9123)	*p* Value	≤ 15 years (n = 1365)	Adults (n = 10,344)	*p* Value	Total (n = 11,709)
Sex ratio (M/F)	–	–		1.1 (726/639)	0.2 (1859/8484)		0.3 (2585/9123)
Mean age	32.6 years	32.7 years	0.743	11.6 years	35.5 years	< 0.001	32.7 years
Median age	31 years	30 years		12 years	32 years		30 years
(range)	(1–98 years)	(2–103 years)		(1–15 years)	(16–103 years)		(1–103 years)
*Age groups*							
≤ 15	726 (28.1%)	369 (4%)	< 0.001	1365 (100%)	–	–	1365 (11.7%)
16–25	315 (12.2%)	2615 (28.7%)	< 0.001	–	2930 (28.3%)	–	2930 (25%)
26–35	473 (18.3%)	2734 (30%)	< 0.001	–	3208 (31%)	–	3208 (27.4%)
36–45	450 (17.4%)	1648 (18.1%)	< 0.001	–	2098 (20.3%)	–	2098 (17.9%)
46–55	271 (10.5%)	751 (8.2%)	< 0.001	–	1022 (9.9%)	–	1022 (8.7%)
56–65	200 (7.7%)	432 (4.7%)	< 0.001	–	632 (6.1%)	–	632 (5.4%)
> 65	150 (5.8%)	304 (3.3%)	< 0.001	–	454 (4.4%)	–	454 (3.9%)
*Reported status*							
Displaced	1763 (68.2%)	6491 (71.1%)	0.003	1121 (82.1%)	7133 (69.1%)	< 0.001	8254 (70.5%)
Resident	809 (31.3%)	2580 (28.3%)	0.001	235 (17.2%)	3155 (30.6%)	< 0.001	3390 (29%)
*Education*							
Illiterate	1407 (54.4%)	7239 (79.4%)	< 0.001	702 (51.4%)	7945 (76.8%)	< 0.001	8647 (73.9%)
Primary	762 (29.5%)	1276 (14%)	0.003	619 (45.4%)	1418 (13.7%)	0.002	2038 (17.4%)
Secondary	330 (12.8%)	472 (5.2%)	0.004	29 (2.1%)	776 (7.5%)	< 0.001	804 (6.9%)
College	52 (2%)	23 (0.3%)	0.031	–	75 (0.7%)	–	75 (0.6%)
University	8 (0.3%)	2(0.02%)	0.568	–	10 (0.1%)	–	10 (0.1%)

Sex missing for 1 patient; Age missing for 3 patients; Self-reported status missing for 65 patients; Education missing for 135 patients. *p* Values are obtained from chi-squared test

### Symptom categories and stress types

The most common MH symptoms were depression (36.8%; n = 4303) and anxiety (33.6%; n = 3931), followed by posttraumatic (15.9%; n = 1867), somatoform (6.6%; n = 770) and psychotic symptomology (2.8%; n = 323) (Table 2). The most prevalent stress experienced was having a relative with a severe medical condition, sexual violence (inside and outside the family), a family member having been killed or “disappeared,” destroyed or lost property, and forced displacement (IDPs)*.* Most stress events occurred more than a year prior to receiving MH services (38.7%; n = 4525), while a minority occurred less than a month (6.2%; n = 721) or a week prior (8.9%; n = 1041) (Table 3).

**Table 2:** Main symptom categories observed in the study population

Symptom category	Male (n = 2580)	Female (n = 9100)	*p* Value	Children ≤ 15 years (n = 1359)	Adults (n = 10,322)	*p* Value	Total (n = 11,681)
Depression	693 (26.9%)	3610 (39.7%)	< 0.001	333 (24.5%)	3970 (38.5%)	< 0.001	4303 (36.8%)
Anxiety	846 (32.8%)	3085 (33.9%)	< 0.001	469 (34.5%)	3462 (33.5%)	< 0.001	3931 (33.7%)
Posttraumatic	414 (4.4%)	1452 (16%)	0.001	271 (19.9%)	1596 (15.5%)	< 0.001	1867 (16%)
Somatoform	258 (10%)	512 (5.6%)	0.001	107 (7.9%)	663 (6.4%)	0.001	770 (6.6%)
Others	162 (6.3%)	183 (2%)	0.003	98 (7.2%)	247 (2.4%)	0.001	345 (3%)
Psychotic	114 (4.4%)	209 (2.3%)	0.003	43 (3.2%)	280 (2.7%)	0.001	323 (2.8%)
Behavioral	73 (2.8%)	34 (0.4%)	0.004	27 (2%)	80 (0.8%)	0.001	107 (0.9%)
Cognitive	20 (0.8%)	15 (0.2%)	0.015	11 (0.8%)	24 (0.2%)	0.001	35 (0.3%)

Information missing for 28 patients. *p* Values are obtained from chi-squared test

**Table 3:** Frequency of stressors

Stressor	Male	Female	*p* Value	Children ≤ 15 years	Adults	*p* Value	Total
Patient or relative with a severe medical condition*	1136 (19.6%)	3839 (20.6%)	0.047	426 (11.1%)	4549 (22%)	< 0.001	4975 (20.3%)
Sexual violence (inside and outside the family)	826 (14.2%)	3473 (18.6%)	< 0.001	857 (22.4%)	3442 (16.7%)	< 0.001	4299 (17.6%)
Other stressors**	1430 (24.6%)	2696 (14.4%)	< 0.001	1013 (26.5%)	3113 (15.1%)	< 0.001	4126 (16.9%)
Family member killed or forced disappeared***	790 (13.6%)	2990 (16%)	0.016	563 (14.7%)	3217 (15.6%)	< 0.001	3780 (15.4%)
Property destroyed or lost	690 (11.9%)	1989 (10.7%)	< 0.001	221 (5.8%)	2458 (11.9%)	< 0.001	2679 (10.9%)
Forced to flee or IDP	440 (7.6%)	1636 (8.8%)	0.1399	329 (8.6%)	1747 (8.5%)	< 0.001	2076 (8.5%)
Other violent events****	318 (5.5%)	1319 (7.1%)	0.003	259 (6.8%)	1378 (6.7%)	< 0.001	1637 (6.7%)
Having a family member arrested	78 (1.3%)	373 (2%)	0.006	74 (1.9%)	377 (1.8%)	< 0.001	451 (1.8%)
Kidnapping	22 (0.4%)	207 (1.1%)	< 0.001	32 (0.8%)	197 (1%)	< 0.001	229 (0.9%)
History of psychiatric disorders	80 (1.4%)	148 (0.8%)	< 0.001	48 (1.2%)	180 (0.9%)	< 0.001	228 (0.9%)

Information missing for 59 patients. *Includes severe medical conditions, family member medical illness, and highly stigmatized diseases stressors. **Includes other stressors, family member died, loss of family income, accidents, unaccompanied minor, and children caretakers neglected stressors. ***Includes family member forced disappeared, and family member(s) killed stressors. ****Includes forced recruitment by armed groups, receive threats, combat experience, incarceration, other physical violence, and torture stressors. *p* Values are obtained from chi-squared test

Women were more likely than men to have suffered sexual violence (SV) (OR: 1.4, *p* < 0.001), although nearly one-fifth of male patients experienced SV as well (14.2%; n = 826). SV was strongly associated with posttraumatic symptomology (OR: 21.2, *p* < 0.001), depression (OR: 3.8, *p* < 0.001), and somatoform symptomology (OR: 1.5, *p* = 0.002). Children who suffered sexual violence were also far more likely than adults to present with posttraumatic (OR: 29.8, *p* < 0.001), depression (OR: 5.8, *p* < 0.001), somatoform (OR: 2.2, *p* = 0.008), and anxiety (OR: 3.8, *p* < 0.001) symptom categories.

We found that patients presenting with posttraumatic or depressive symptomology had a higher probability of having experienced conflict-related violence. Patients who suffered combat experience (OR: 19.7, *p* < 0.001), had a family member killed (OR: 19.3, *p* < 0.001), or had received threats (OR: 16.3, *p* < 0.001) were more likely to present with posttraumatic symptomology. Forced displacement (IDPs) (OR: 4.4, *p* < 0.001), incarceration (OR: 4.6, *p* = 0.015), and having received threats (OR: 1.3, *p* < 0.001) were associated with depression. Posttraumatic and depression-related symptoms were associated with forced recruitment by armed groups (OR: 18.4, *p* = 0.003) (OR: 8.7, *p* = 0.044) and torture (OR: 11.9, *p* = 0.038) (OR: 1.4, *p* = 0.027), respectively. Patients presenting with posttraumatic (OR: 6.5, *p* < 0.001) and psychotic (OR: 3.2, *p* < 0.001) symptom categories had a higher probability of reporting that their primary stress event occurred more than one year before presenting to care (Tables 4, 5).

**Table 4:** factors associated with presenting a concrete disorder for adults (*p* Value and OR from the multivariable logistic regression models)

	Somatoform		Anxiety		Posttraumatic		Depression		Psychotic	
	OR [95% IC]	*p* Value	OR [95% IC]	*p* Value	OR [95% IC]	*p* Value	OR [95% IC]	*p* Value	OR [95% IC]	*p* Value
*Age by groups*										
16–25	0.48 [0.37–0.62]	< 0.001	1.88 [1.46–2.42]	< 0.001	0.48 [0.35–0.66]	< 0.001	0.80 [0.63–1.03]	0.088	0.52 [0.33–0.81]	0.004
26–35	0.46 [0.36–0.59]	< 0.001	1.86 [1.45–2.38]	< 0.001	0.67 [0.49–1.90]	0.009	0.75 [0.57–0.96]	0.027	0.33 [0.21–0.53]	< 0.001
36–45	0.55 [0.43–0.71]	< 0.001	1.47 [1.14–1.89]	0.003	0.84 [0.62–1.14]	0.273	0.85 [0.66–1.10]	0.233	0.39 [0.25–0.63]	< 0.001
46–55	0.71 [0.54–0.94]	0.002	1.28 [0.97–1.69]	0.076	1.27 [0.92–1.77]	0.138	1.00 [0.76–1.32]	0.976	0.33 [0.18–0.57]	< 0.001
56–65	0.91 [0.67–1.20]	0.541	1.21 [0.89–1.63]	0.216	1.00 [0.69–1.43]	0.998	0.78 [0.57–1.05]	0.107	0.46 [0.26–0.83]	0.010
> 65	Ref	Ref	Ref	Ref	Ref	Ref	Ref	Ref	Ref	Ref
*Gender*										
Male	1.67 [1.46–1.91]	< 0.001	1.25 [1.08–1.44]	0.002	Ref	Ref	0.81 [0.72–0.92]	0.002	2.40 [1.81–3.14]	< 0.001
Female	Ref	Ref	Ref	Ref	1.48 [1.24–1.78]	< 0.001	Ref	Ref	Ref	Ref
*Education*										
Illiterate	3.31 [2.00–5.47]	< 0.001	–	–	–	–	–	–	–	–
Primary	3.03 [1.81–5.08]	< 0.001	–	–	–	–	–	–	–	–
Secondary	3.12 [1.84–5.27]	< 0.001	–	–	–	–	–	–	–	–
College	2.40 [1.16–4.97]	0.018	–	–	–	–	–	–	–	–
University	2.85 [0.69–11.7]	0.147	–	–	–	–	–	–	–	–
Unknown	Ref	Ref	–	–	–	–	–	–	–	–
*Event date*										
> 1 year	0.85 [0.69–1.04]	0.116	0.43 [0.34–0.55]	< 0.001	6.49 [3.97–10.6]	< 0.001	0.84 [0.68–1.03]	0.105	3.17 [1.55–6.50]	< 0.001
4–12 months	1.08 [0.84–1.39]	0.531	0.58 [0.43–0.78]	< 0.001	5.43 [3.20–9.23]	< 0.001	0.97 [0.75–1.25]	0.840	1.57 [0.66–3.71]	0.304
1–3 months	1.25 [0.97–1.61]	0.073	0.48 [0.36–0.65]	< 0.001	5.11 [3.00–8.75]	< 0.001	0.96 [0.74–1.25]	0.794	1.96 [0.76–5.03]	0.160
1–4 weeks	1.49 [1.17–1.90]	< 0.001	0.77 [0.58–1.02]	0.077	1.88 [1.06–3.34]	0.031	0.70 [0.54–0.90]	0.007	1.65 [0.64–4.27]	0.295
4–7 days	1.34 [1.03–1.74]	0.025	0.71 [0.52–1.27]	0.066	2.41 [1.34–4.30]	0.003	0.96 [0.73–1.25]	0.779	1.61 [0.56–4.60]	0.370
1–3 days	Ref	Ref	Ref	Ref	Ref	Ref	Ref	Ref	Ref	Ref

*Reference category (Ref.).* Somatoform model: Pearson's Goodness-of-Fit Test = 0.1526; Pseudo R^2^ = 0.028. Anxiety model: Pearson's Goodness-of-Fit Test = 0.225; Pseudo R^2^ = 0.032. Posttraumatic model: Pearson's Goodness-of-Fit Test = 0.467; Pseudo R^2^ = 0.051. Depression model: Pearson's Goodness-of-Fit Test = 0.412; Pseudo R^2^ = 0.072. Psychotic model: Pearson's Goodness-of-Fit Test = 0.566; Pseudo R^2^ = 0.052

**Table 5:** Factors associated with presenting a concrete disorder for adults (*p* Value and OR from the multivariable logistic regression models)

	Somatoform		Anxiety		Posttraumatic		Depression	
	OR [95% IC]	*p* Value	OR [95% IC]	*p* Value	OR [95% IC]	*p* Value	OR [95% IC]	*p* Value
*Stressors*								
Severe medical conditions	1.73 [0.96–1.53]	< 0.001	1.49 [1.33–1.68]	< 0.001	0.48 [0.41–0.57]	< 0.001	0.59 [0.52–0.66]	< 0.001
Unwanted pregnancy	–	–	–	–	–	–	3.94 [1.97–7.88]	0.027
Family member medical illness	0.67 [0.58–0.83]	0.002	3.21 [2.76–3.72]	< 0.001	–	–	0.66 [0.58–0.75]	< 0.001
Sexual violence	1.75 [1.47–2.08]	< 0.001	–	–	21.19 [11.8–37.8]	< 0.001	3.83 [2.92–5.01]	< 0.001
Combat experience	–	–	–	–	19.72 [6.54–59.4]	< 0.001	–	–
Incarceration	–	–	–	–	6.29 [1.23–32.2]	0.027	4.57 [1.34–15.2]	0.015
Kidnapping	0.36 [0.26–0.51]	< 0.001	–	–	8.88 [4.34–18.2]	< 0.001	0.62 [0.46–0.84]	< 0.001
Domestic violence	0.46 [0.38–0.57]	< 0.001	0.61 [0.50–0.75]	< 0.001	0.51 [0.38–0.67]	< 0.001	1.95 [1.65–2.31]	< 0.001
Receive threats	–	–	1.55 [1.22–2.34]	0.007	16.28 [8.16–32.5]	< 0.001	1.30 [1.10–1.53]	< 0.001
Family member(s) killed	–	–	0.42 [0.38–0.47]	< 0.001	19.25 [10.8–34.2]	< 0.001	1.15 [1.04–1.29]	< 0.001
Unaccompanied minor	–	–	–	–	27.61 [9.28–82.1]	< 0.001	–	–
Caretaker’s neglect (to children)	–	–	–	–	4.78 [1.71–13.4	0.003	2.20 [1.17–4.13]	0.014
Property destroyed or lost	2.02 [1.84–2.24]	< 0.001	1.89 [1.67–2.17]	< 0.001	6.76 [3.73–12.3]	< 0.001	1.35 [1.22–1.50]	< 0.001
Loss of family income	1.78 [1.42–2.05]	< 0.001	1.38 [1.21–1.56]	< 0.001	9.94 [5.25–18.8]	< 0.001	1.64 [1.16–2.32]	0.005
Family member natural died	–	–	0.86 [0.76–0.98]	0.025	7.24 [3.88–13.5]	< 0.001	1.42 [1.14–1.45]	< 0.001
Having a family member arrested	–	–	1.94 [1.17–3.22]	0.010	6.23 [2.79–13.9]	< 0.001	1.40 [1.12–1.76]	< 0.001
Family member forced disappeared	1.58 [1.83–2.23]	< 0.001	2.17 [1.48–3.17]	< 0.001	5.65 [2.84–11.2]	< 0.001	1.77 [1.54–2.04]	< 0.001
Forced to flee or IDP	2.00 [1.79–3.04]	0.002	1.71 [1.48–1.95]	< 0.001	6.01 [2.82–12.8]	< 0.001	4.41 [2.79–6.95]	< 0.001
Other physical violence	–	–	1.26 [1.01–1.57]	0.038	–	–	2.30 [1.27–4.17]	0.006
Torture	–	–	–	–	11.86 [1.14–123]	0.038	1.42 [1.15–28.9]	0.027
Recruitment by armed groups	–	–	–	–	18.40 [2.77–122]	0.003	8.74 [1.22–79.3]	0.044
Others	Ref	Ref	Ref	Ref	Ref	Ref	Ref	Ref

*Reference category (Ref.). Somatoform model:* Pearson's Goodness-of-Fit Test = *0.269; Pseudo R*^*2*^ = *0.042. Anxiety model:* Pearson's Goodness-of-Fit Test = *0.272; Pseudo R*^*2*^ = *0.062. Posttraumatic model:* Pearson's Goodness-of-Fit Test = *0.387; Pseudo R*^*2*^ = *0.156. Depression model:* Pearson's Goodness-of-Fit Test = *0.150; Pseudo R*^*2*^ = *0.090*

In children, multivariable analysis showed that exposure to other types of physical violence (i.e., events that were not torture or SV) (OR: 38.2, *p* = 0.002) was associated with posttraumatic symptoms. Destroyed or lost property (OR: 26, *p* < 0.001) and the arrest of a family member (OR: 13.8, *p* < 0.001) was associated with depression. Children who were forcibly displaced (IDPs) (OR: 25.2, *p* < 0.001) (OR: 11.8, *p* < 0.001), kidnapped (OR: 11.1, *p* = 0.01) (OR: 16.2, *p* < 0.001), or had a family member killed (OR: 15.8, *p* < 0.001) (OR: 13.6, *p* < 0.001) were more likely to present with posttraumatic and depressive symptom categories, respectively.

### Severity of illness

Most patients presented to care with mild or moderate illness (45.1%; n = 5277 and 32.8%; n = 3839, respectively), with only 6.8% (n = 803) categorized as severe at baseline. In multivariable analysis, severe patients were more likely to have experienced a stressor 4 and 12 months before their baseline visit (OR: 1.8, *p* = 0.049). The only specific stress associated with higher severity scores was being an unaccompanied minor (OR: 7.6, *p* = 0.003).

### Patient outcomes

Among the 6046 patients with recorded information at the final session of their MH treatment, a little more than half were discharged voluntarily (60.5%; n = 3650), sometimes after a single session intervention (22.7%; n = 1370), or because they were no longer able to be contacted (7%; n = 422), moved to other location (4.2%; n = 253), or were referred to other services (2.8%; n = 166). CGI measurement at the end of treatment showed that 91.5% (n = 3,279) of patients’ conditions were either “much” or “very much” improved (33.8%; n = 1211, 57.7%; n = 2068, respectively). By the MHGS scale for adults at closure, 72.6% of patients (n = 2451) rated improvement, with only 1.1% (n = 36) reporting a worsening of their symptoms. Far fewer children (45.2%; n = 239) improved overall, however, with nearly half (53.5%; n = 283) showing no change at the conclusion of their care, though similarly few (1.3%; n = 7) reported a worsening condition.

Multivariable analysis showed that some factors were associated with improvement using both the CGI-I and MHGS scales. Patients with somatoform (CGI-I OR: 1.5, *p* < 0.001; MHGS OR: 1.2, *p* = 0.002), posttraumatic (CGI-I OR: 1.3, *p* = 0.003; MHGS OR: 1.5, < 0.001), and depression symptom categories (CGI-I OR: 1.3, *p* < 0.001; MHGS OR: 1.2, *p* = 0.001) were more likely to improve, as were more severe patients at enrollment (OR: 1.8, *p* < 0.001). Similar associations were found in children using the CGI-I scale (somatoform symptoms OR: 2.3, *p* < 0.001; posttraumatic symptoms OR: 2, *p* < 0.001; anxiety symptoms OR: 1.6, *p* = 0.001; and depression OR: 1.5, *p* = 0.002) were more likely to have better improvement outcomes. The MHGS scale in children was somewhat different, with only depressive symptomology (OR: 1.4, *p* = 0.015) showing a similar association.

### Number of sessions and improvement

**Fig. 1:**
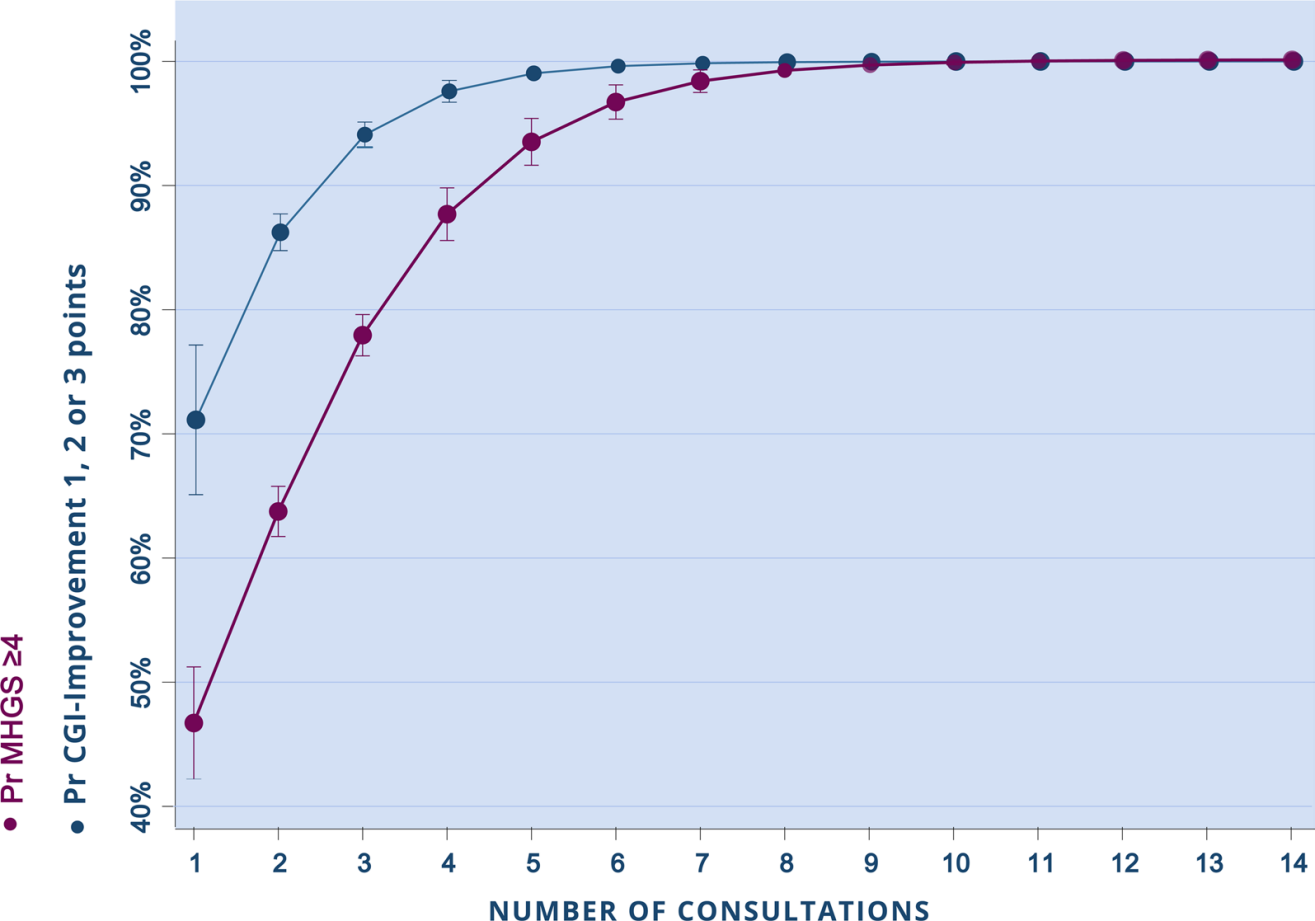
Probability of presenting an improvement outcome according to MHGS and CGI-Improvement scales (y-axis) by the number of consultations received (x-axis) for patients categorized as mild or moderate

**Fig. 2:**
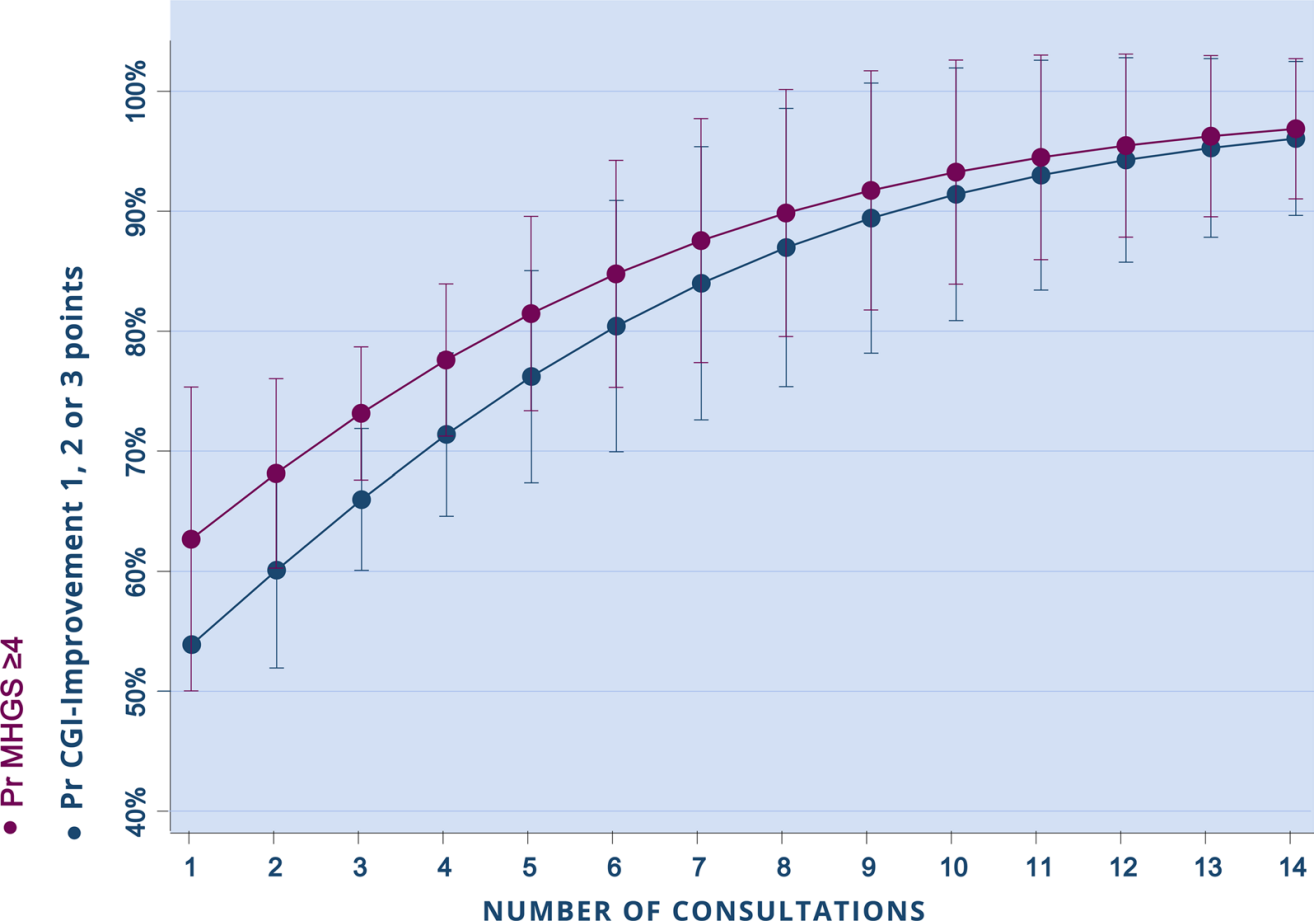
Probability of presenting an improvement outcome according to MGHS and CGI-Improvement scales (y-axis) by the number of consultations received (x-axis) for patients categorized as severe

The mean (SD) number of sessions for all patients (n = 11,709) was 1.8 (0.3) and the median number was 2 (IQR: 7.5). Among patients with a final consultation recorded, 64.6% participated in 2 or more sessions (n = 3911). For every additional counseling session received, the patient’s odds of improvement increased (CGI-I OR: 2.5, *p* < 0.001; MHGS OR: 2, *p* < 0.001). Therefore, having three sessions increased the patient’s likelihood of improving by 95% (using the CGI-I scale) and 78% (using the MHGS scale) (Fig. 1). Among the initially severe patients with a final consultation recorded, slower improvement per session was noted using both the CGI-I scale (OR: 1.3, *p* = 0.019), and the MHGS (OR: 1.3, *p* = 0.041), with having received 6 sessions increasing the probability of improvement by 81% (CGI-I) and 85% (MHGS) (Fig. 2).

## Discussion

Routinely collected clinical data from active MHPSS sites allowed investigators to have a “real world” view of the impact of MHPSS care in extremely challenging environments. The nearly 12,000 conflict affected patients in our study demonstrated that the type and severity of trauma and symptoms they experienced had a direct effect on their ability to improve while under the care of a MH professional, as did the duration of the MH services they received.

We found that, despite a high rate of LTFU among these patients (not unlike other MH programs in conflict settings), receiving even two sessions with a MH professional proved to be of enormous benefit [10]. One-third of patients participated in only a single therapeutic session, either because they were better suited to a single-session intervention strategy or because of LTFU. Additionally, in Nigeria, the affected population’s needs were numerous and the security situation volatile, making short psychological interventions a necessity. Single-session psychological interventions have proven effectiveness and feasibility in humanitarian settings, and psychotherapeutic improvement is known to often occur during the first sessions [12, 13, 19–21]. Yet in recent years, some WHO guidance has encouraged a standard of at least five sessions (including enrollment and final consultations) [22], though it remains unclear why a five-session minimum should apply to most patients. Indeed, the question seems unsettled, with some studies finding that a four-session threshold is appropriate, while another randomized trial found that 38% of mild depression patients’ symptoms were relieved by session two. Debate persists over whether and how the number of sessions influences the result of therapy [23], and our results further question the utility of five-session minimums for all patients and lead us to believe that more single, three, and six-session strategies should be developed to better fit specific population needs. Long-term interventions and pharmacological treatment should also be available for patients with chronic mental illness, and MHPSS programs should evaluate clinical severity in order to apply tailored interventions.

As with other cohorts, a large proportion of patients were exposed to SV and other conflict/violence-related stress, and SV was strongly associated with posttraumatic, somatoform, and depression symptoms [4, 5, 10, 24, 25]. Our results also revealed that, though most SV survivors were female, nearly one-fifth of men had also experienced this type of violence (a likely underestimate because of the extreme stigma surrounding male SV). Further research is needed to address gendered differences in access to MHPSS care, to better understand the MH consequences of SV in male survivors, and to remove the barriers to care that they face [26–28].

Combat experience, having a family member killed, forced recruitment by armed groups, having received threats, having destroyed or lost property, and having been tortured showed a strong relationship with posttraumatic and depression symptomology, consistent with other studies [4, 8, 10, 29, 30]. Also in line with other studies, we found that most PTEs occurred more than a year before consultation, and that these patients were more likely to present with posttraumatic or psychotic symptomology (and less likely to present with anxiety) [10]. Unaccompanied minors were more likely to present with more severe symptoms (minors who experience caretaker neglect have been shown to have high distress levels when previously studied) [10], highlighting the importance of detecting and supporting unaccompanied minors, and prioritizing them in MHPSS programs.

According to both of the scales used in the study, we found that a large majority of patients improved during the course of their MH care, at rates similar to other research (70–90%) [4–6, 31, 32]. Both the CGI-I and MHGS scales used are culturally validated and trans-diagnostic, an essential need for MHPSS programs in humanitarian settings where task-shifting models are frequently used and non-specialist staff may provide MH care.

Patients in our cohort, like other conflict-affected populations, frequently struggled with depression, anxiety, and posttraumatic stress symptomology [4–6]. Our study showed that patients who presented with these symptoms, as well as those who were initially more severe, were more likely to improve. One previous study showed that lower function at enrollment was significantly associated with improvement in psychological distress [10].

### Limitations

This study is limited by its specific context and care provider (MSF); generalizations should be made with caution. The study design only investigates associations between baseline risk factors (stress type, symptom severity). Results may be confounded by external and environmental factors that occurred outside of counseling sessions. Notwithstanding training and regular supervision, the routinely collected data used in this study is subject to human and data entry error. Given its methodology, the study’s high LTFU rate potentially indicates that many patients’ care was truncated without closure and that their data (especially from final therapy sessions) are missing. For this reason, only 52% of the patients with a final consultation were included in the regression analysis, and characteristics and trends among the excluded may differ from those reported in our results. Despite having been internationally recognized across multiple settings, the MHGS scale used in the study has not undergone extensive testing and validation in Nigeria or West Africa. However, investigators assessed its appropriateness in the context by conducting abbreviated cross-cultural validation exercises in Pulka and Gwoza LGAs before being more widely implementation. Lastly, as the MHPSS program in Nigeria was ongoing at the time of analysis, some patients with chronic and severe mental disorders were still undergoing treatment and were thus not included in the mean average sessions or outcomes analysis.

## Conclusion

Our study suggests that patients with mild or moderate MH conditions improve after three sessions, while patients with severe symptoms need at least six. Specific types of stress and symptom severity affected the number of sessions needed to achieve successful outcomes, and some stress types (especially torture or having a relative killed) were specifically linked to PTSD and depression. Classifying patient stress and severity can identify the appropriate duration of care needed and reduce the risk of patients defaulting from care. MH professionals serving patients in similarly violent or conflict-affected settings should focus on identifying which patients could benefit from a single-session intervention strategy versus those who need a brief (three or six sessions) or long-term/chronic interventions. Furthermore, newer, three-session formats of low-intensity psychological care merit being developed and tested.

Humanitarian crises are a major global health challenge and have profound repercussions for the affected population’s MH. MHPSS programs alleviate suffering, reduce the psychological consequences of war and violence, and constitute a key component of the humanitarian crisis response [2, 3]. Given the enormous needs and lack of specialized resources, psychological interventions in humanitarian settings should continue to evolve and adapt.

## Data Availability

The datasets generated and analyzed during the current study are not publicly available due to confidentiality, and access is restricted for non-MSF staff. However, it is available from the corresponding author on reasonable request.

## Abbreviations

CGI-I: Clinical Global Impression-Improvement scale
CGI-S: Clinical Global Impression-Severity scale
DSM-IV: Diagnostic and Statistical Manual of Mental Disorders-IV
ICD-10: International Disease Classification 10 Manual
IDP: Internally displaced persons
LGA: Local government areas
LTFU: Loss to follow up
MSF: Médecins Sans Frontières
MH: Mental health
mhGAP: Mental Health Gap Action Programme
MHGS: Mental Health Global State scale
MHPSS: Mental health and psychosocial support
MNS: Mental, neurological, and substance use
PTE: Potentially traumatic events
PTSD: Posttraumatic stress disorder
SV: Sexual violence
WHO: World Health Organization
